# Kinesin light chain 4 as a new target for lung cancer chemoresistance via targeted inhibition of checkpoint kinases in the DNA repair network

**DOI:** 10.1038/s41419-020-2592-z

**Published:** 2020-05-26

**Authors:** Jeong-Hwa Baek, Hong Shik Yun, Ju-Young Kim, Janet Lee, Yeon-Joo Lee, Chang-Woo Lee, Jie-Young Song, Jiyeon Ahn, Jong Kuk Park, Jae-Sung Kim, Kee-Ho Lee, Eun Ho Kim, Sang-Gu Hwang

**Affiliations:** 1 Radiation Biology Research Team, Research Center, Dongnam Institute of Radiological and Medical Sciences, Busan, 46033 Republic of Korea; 2 Radiation Oncology Branch, National Cancer Institute, NIH, Bethesda, MD USA; 3 Division of Radiation Biomedical Research, Korea Institute of Radiological & Medical Sciences, Seoul, 01812 Korea; 4 Department of Molecular Cell Biology, Sungkyunkwan University School of Medicine, Suwon, 440-746 Korea; 5 Department of Biochemistry, School of Medicine, Daegu Catholic University, 33, 17-gil, Duryugongwon-ro, Nam-gu, Daegu, Korea

**Keywords:** Tumour biomarkers, Tumour biomarkers, Tumour biomarkers, Tumour biomarkers, Prognostic markers

## Abstract

The poor therapeutic efficacy of non-small cell lung cancer (NSCLC) is partly attributed to the acquisition of chemoresistance. To investigate the mechanism underlying this resistance, we examined the potential link between kinesin light chain 4 (KLC4), which we have previously reported to be associated with radioresistance in NSCLC, and sensitivity to chemotherapy in human lung cancer cell lines. KLC4 protein levels in lung cancer cells correlated with the degree of chemoresistance to cisplatin treatment. Furthermore, *KLC4* silencing enhanced the cytotoxic effect of cisplatin by promoting DNA double-strand breaks and apoptosis. These effects were mediated by interaction with the checkpoint kinase CHK2, as *KLC4* knockdown increased CHK2 activation, which was further enhanced in combination with cisplatin treatment. In addition, *KLC4* and *CHEK2* expression levels showed negative correlation in lung tumor samples from patients, and *KLC4* overexpression correlated negatively with survival. Our results indicate a novel link between the KLC4 and CHK2 pathways regulating DNA damage response in chemoresistance, and highlight KLC4 as a candidate for developing lung cancer-specific drugs and customized targeted molecular therapy.

## Introduction

Lung cancer is one of the main reasons of cancer-related deaths worldwide, and 80% cases of lung cancer are non-small cell lung cancer (NSCLC) with poor prognosis at diagnosis and limited therapeutic efficiency^1,2^. Current research directions for lung cancer treatment are inclusive of immunotherapy, which makes it possible for the body’s immune system to attack the tumor cells, epigenetics, and new combinations of chemotherapy and radiotherapy, both on their own and collectively. Many of these new treatments work through immune checkpoint blockade, thus disrupting cancer’s ability to evade the immune system^3,4^. Targeted therapy and immunotherapy are known to significantly prolong the 5-year survival, and more than 17% patients can survive for 5 years after being diagnosed with advanced stage NSCLC^5^. However, chemoresistance for lung cancer treatment remains an important barrier to treatment efficacy^6,7^. Lung cancer is well-known to have high recurrence rate, as lung cancer cells cannot be entirely removed using conventional chemotherapeutics owing to the development of drug resistance^8^. Cisplatin can cause interstrand and intrastrand crosslinks between purine bases; therefore, it is widely used for treating solid tumors^9^. However, chronic treatment with cisplatin can induce a chemoresistant phenotype. Therefore, identifying the genes underlying this chemoresistant phenotype is critical for better understanding of the molecular pathogenesis of NSCLC and for developing gene-targeted therapies aimed at treating chemoresistant cancer. Furthermore, elucidating the molecular mechanisms of cisplatin resistance will assist in establishing effective prognostic biomarkers and enhancing the efficiency of related therapeutic interventions.

Toward this, extensive investigations have focused on uncovering the molecular mechanism contributing to the initiation and progression of NSCLC, resulting in the development of diverse novel targeted agents such as epidermal growth factor receptor tyrosine kinase inhibitors, which are more efficient than chemotherapy in patients with epidermal growth factor receptor-mutated tumors^10^. Despite numerous experimental studies in this field, studies focusing on the mechanisms of resistance based on clinical therapy are scarce and generally include only a small number of patients.

Checkpoint kinases (CHK1 and CHK2) have recently emerged as potential new targets for cancer therapy to enhance response to genotoxic drugs. CHK1 and CHK2 play critical roles in the DNA damage response (DDR) network^11^, and DNA repair pathways are important for resistance to DNA-damaging cytotoxic therapy and radiation^12^. Cellular defenses against DNA damage are regulated by multiple checkpoints that enable cell cycle arrest, DNA repair, or apoptosis in case of extensive DNA damage^13,14^. CHK2 plays particularly vital roles in DDR via signaling of the ATM-CHK2-P53 pathway, and in regulating cell cycle checkpoints, including the G2/M checkpoint^15,16^. CHK2 phosphorylates multiple target proteins that are involved in oncogenesis, including p53, CDC25A, CDC25C, BRCA1, E2F1, and MDC1^11^. Support for the role of CHK2 in cancer comes from rare germline or somatic mutations in *CHK2* in certain human familial cancers and several tumor types, and from its important role in oncogene-induced senescence^16^. Furthermore, several reports indicate the advantage of CHK2 inhibition in inducing tumor killing in response to genotoxic drugs^15^. CHK2 has been verified as a tumor suppressor, and is mutated or depleted in several cancers, including breast, colon, bladder, ovarian, and prostate carcinomas^17,18^. In addition, low level of CHK2 in lung cancers was suggested to contribute to chemo-radiation resistance^19^.

Recently, we identified several proteins, including kinesin light chain 4 (KLC4), to be involved in the radioresistance of NSCLC^20^. However, the regulatory mechanism linking KLC4 expression and sensitivity to chemotherapy or radioresistance in lung cancer remains unclear. We first investigated whether KLC4 expression and sensitivity to chemotherapy or radioresistance in lung cancer cell lines treated with cisplatin or other common chemotherapy drugs were related. We further hypothesized that KLC4 may be involved in the DDR via interaction with CHK1/2 to drive chemoresistance. Therefore, we investigated the effect of *KLC4* knockdown on CHK1/2 activation, cytotoxicity, and DNA damage induction by cisplatin. Our study highlights a new candidate for the development of lung cancer-specific drugs and customized targeted molecular therapy.

## Results

### KLC4 regulated chemoresistance in lung cancer cells

**Fig. 1:**
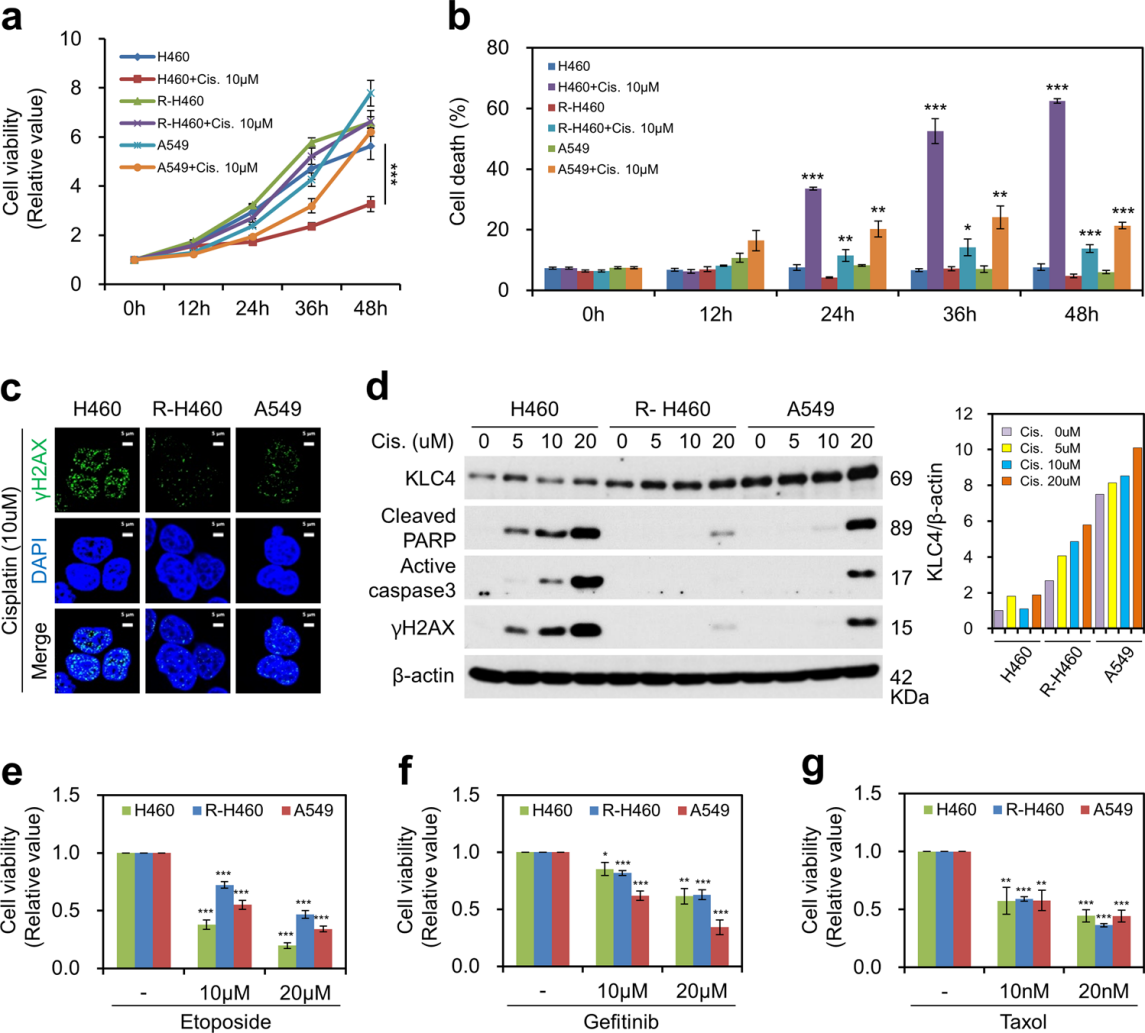
KLC4 was related to chemoresistance in vitro. **a** Cell viability was measured every 12 h after treatment with 10 μM cisplatin. **b** Death of cells treated with or without 10 μM cisplatin during 12 h intervals. **c** After cisplatin treatment for 24 h, the cells were fixed with 4% paraformaldehyde and immunostained using an antibody targeting γH2AX (DNA damage marker); DNA was visualized using DAPI staining. **d** Protein levels of KLC4, cleaved PARP, and active caspase-3 were determined using western blotting after cisplatin treatment. **e**–**g** Cells treated with etoposide, gefitinib, or Taxol, respectively. Twenty-four hours after the drug treatments, the cells were analyzed using cell viability assay, followed by ELISA.

We first evaluated the anticancer drug resistance of the lung cancer cell lines, H460 with lower KLC4 expression, and R-H460 and A549 with higher KLC4 expression than that of H460 cells. We assessed the effect of cisplatin treatment on cell growth and proliferation of the three lung cancer cell lines. The cell viability assay showed that 10 μM cisplatin (treated for 0, 12, 24, 36, and 48 h) significantly (*P* < 0.001) inhibited the growth of H460 cells (Fig. 1a).

As shown in Fig. 1b, cisplatin-induced considerable apoptosis in H460 cells; however, compared with that in H460 cells, apoptosis was not significantly increased in R-H460 and A549 cells. To investigate whether cisplatin as a DNA damage inducer altered double strand break (DSB) repair, we attempted to validate DDR according to KLC4 expression in the lung cancer cell lines. We monitored the formation of γ-H2AX foci in the cisplatin-treated cells and observed an increase in the number of γ-H2AX foci after 24 h (Fig. 1c). Furthermore, compared with those in R-H460 and A549 cells, cisplatin significantly increased the number of γ-H2AX foci in H460 cells (Fig. 1c). Western blotting further showed that KLC4 protein levels in human lung cancer cell lines (H460, R-H460, and A549 cells) correlated with the tendency of chemoresistance (Fig. 1d). H460 cells were more sensitive to cisplatin than R-H460 and A549 cells. These results indicated a correlation between increase in KLC4 expression and chemoresistance. Several lines of evidence suggest that lung cancer cells are resistant to multiple therapeutic agents^21^. Therefore, we further assessed the inhibition of cell viability of a spectrum of chemoresistant lung cancer cells (H460, R-H460, and A549 cells) treated with conventional chemotherapeutic drugs that are well-known inducers of apoptosis, including etoposide (DNA-damaging agent), gefitinib (EGFR inhibitor), and Taxol (microtubule inhibitor), for 24 h^22^. Similar to the effect of cisplatin, only etoposide, but not gefitinib or Taxol, correlated with the expression level of KLC4 and inhibition of cell viability (Fig. 1e–g). Thus, KLC4 is expected to be involved in the positive resistance to DNA damage-related anticancer drugs.

### *KLC4* knockdown induced growth inhibition and apoptosis in cisplatin- or etoposide- treated lung cancer cells

**Fig. 2:**
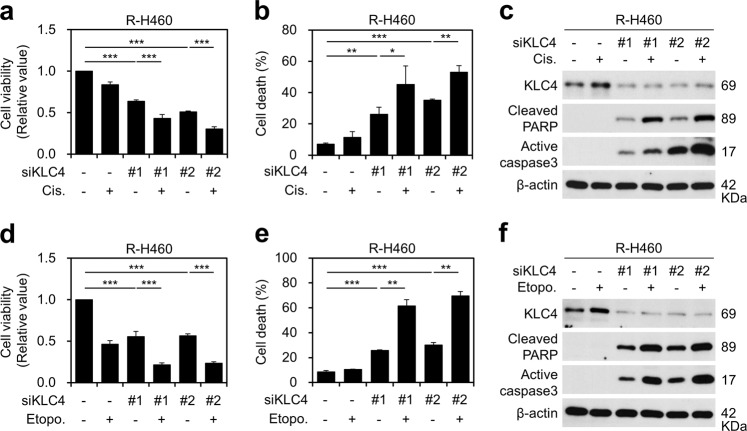
*KLC4* depletion reversed chemoresistance in lung cancer cells. **a** Viability of R-H460 cells treated with or without 10 μM cisplatin after transfection with siCON (negative control) or siKLC4. **b** Cell death in R-H460 cells [treated as described in (**a**)] using annexin V/propidium iodide staining. **c** Protein levels of KLC4, cleaved PARP, and active caspase-3 (cell death marker) as determined using western blotting. **d** Viability of R-H460 cells treated with or without 10 μM etoposide after transfection with siCON or siKLC4. **e**−**f** R-H460 cells were treated with or without 10 µM etoposide after transfection with *KLC4* siRNA. Cell death was measured 48 h after treatment using annexin V/propidium iodide staining (**e**), and western blotting (**f**).

To further investigate the effects of *KLC4* in regulating the fate of lung cancer cells treated with anticancer drugs, the gene was silenced via RNA interference using specific siRNA targeting *KLC4*. As illustrated in Fig. 2c and Supplementary Fig. 1c, *KLC4* was successfully knocked down in R-H460 and A549 cells after transfection with the *KLC4* siRNA. Furthermore, compared with that observed with *KLC4* silencing alone in R-H460 and A549 cells, the combination of *KLC4* silencing with cisplatin treatment decreased cell viability (Fig. 2a, Supplementary Fig. 1a). The anchorage-dependent colony forming assay showed that *KLC4* siRNA plus cisplatin significantly (*P* < 0.001) decreased the number of colonies formed in A549 cells (Supplementary Fig. 2a). Compared with that observed with *KLC4* siRNA treatment alone, knockdown of *KLC4* in combination with cisplatin also increased lung cancer cell death, as was evident from the evaluation of apoptosis using flow cytometry (Fig. 2b, Supplementary Fig. 1b). In addition, the levels of cleaved PARP and active caspase-3 were higher in *KLC4* siRNA-transfected cells combined with cisplatin treatment than in untreated siRNA-transfected cells (Fig. 2c, Supplementary Fig. 1c). Similarly, compared with that observed with *KLC4* siRNA treatment alone in R-H460 and A549 cell lines, the combination of etoposide with *KLC4* siRNA treatment significantly inhibited cell viability and cell death (Fig. 2d–f, Supplementary Fig. 2d–f). These results showed that *KLC4* knockdown enhanced the cytotoxicity of cisplatin and etoposide, indicating *KLC4* as a novel chemoresistance gene in lung cancer.

### Knockdown of *KLC4* in lung cancer cells enhanced cisplatin or etoposide-induced DNA damage and regulated non-homologous end joining (NHEJ) repair

**Fig. 3:**
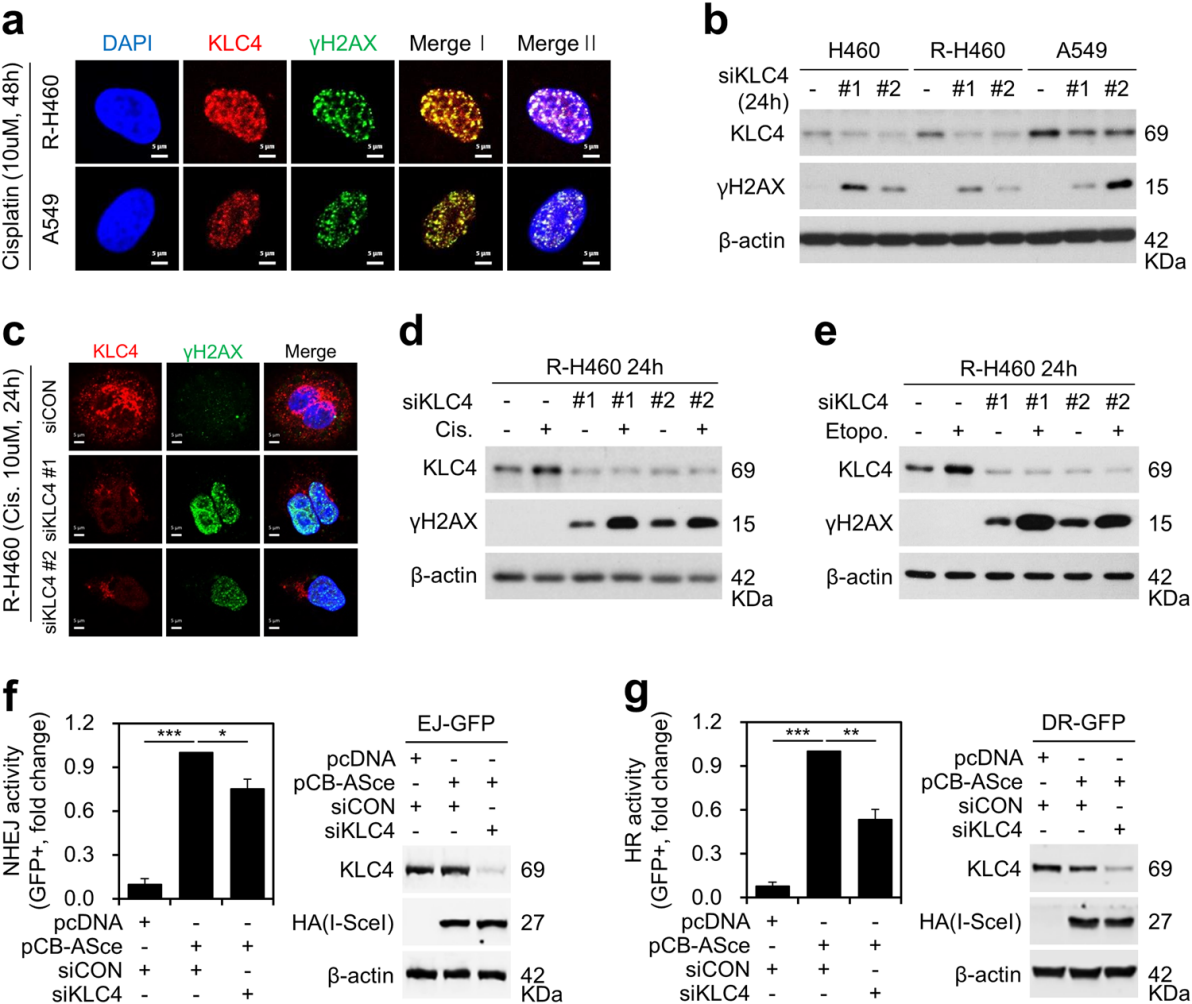
Downregulation of *KLC4* induced DNA damage response. **a** Cells were fixed with 4% paraformaldehyde and stained using antibodies targeting KLC4 and γH2AX; DNA was visualized using DAPI staining. **b** Protein levels of KLC4 and γH2AX were determined using western blotting after transfection with *KLC4* siRNA. **c** R-H460 cells were treated with 10 µM cisplatin after transfection with *KLC4* siRNA for 24 h. Cells were fixed and immunostained using antibodies targeting KLC4 and γH2AX. **d**, **e** R-H460 cells were pre-treated with 10 µM cisplatin (d) or 10 µM etoposide (**e**) and transfected with siKLC4. The cell lysates were prepared and used for immunoblotting with antibodies against KLC4 and γH2AX. **f**, **g** Reporter cells were transfected with *KLC4* siRNA and pCB-ASce vector (HA tagged I-SceI expression). GFP-positive cells were counted using flow cytometry in the U2OS EJ-GFP cell (**f**) or the U2OS DR-GFP cell (**g**).

Cisplatin can lead to the intracellular accumulation of DNA DSBs and γ-H2AX protein, which is responsible for cell apoptosis^23^. Furthermore, etoposide also induces DNA damage in cells by interacting with the nuclear enzyme topoisomerase II^24^. Therefore, we further investigated the effect of cisplatin and/or KLC4 expression on γ-H2AX level, which is the Ser139 phosphorylated form of H2AX and a recognized marker of DNA damage^25^. The immunofluorescence images clearly illustrated that KLC4 colocalized with the cisplatin-induced foci of γ-H2AX in R-H460 and A549 cells (Fig. 3a). Inhibition of KLC4 expression in the three types of lung cancer cell lines increased the expression level of γ-H2AX (Fig. 3b). We detected a significant increase in γ-H2AX foci in the *KLC4* siRNA-transfected R-H460 and A549 cells, which gradually increased to the initial level after additional cisplatin treatment (Fig. 3c, Supplementary Fig. 3a). Consistently, transfection of siKLC4, combined with cisplatin or etoposide, markedly enhanced the accumulation of γ-H2AX at the protein level (Fig. 3d, e, Supplementary Fig. 3b, c), indicating that *KLC4* depletion may considerably increase the genotoxic effect of cisplatin and etoposide in aggravating DNA damage in lung cancer cells. Furthermore, in agreement with previous results, *KLC4* depletion maintained DNA damage, and thus enhanced the radiosensitivity of the cells (Supplementary Fig. 3d). Subsequently, we used the reporter systems to study non-homologous end joining (NHEJ) and homologous recombination (HR) activity as the primary DNA DSB repair pathways^26^. Using U2OS EJ-GFP cells harboring a single chromosomally integrated copy of the pimEJ5-GFP construct^27–29^, the percentage of GFP- positive cells was considerably reduced in cells that were treated with *KLC4* siRNA after exogenous expression of HA tagged I-SceI, which, in turn, suggested the suppression of the NHEJ repair pathway after *KLC4* knockdown (Fig. 3f). Similar to the result of the EJ reporter assay, a considerable change in the GFP-positive rate was detected after silencing *KLC4* in the U2OS cells with stable transfection of the pHRPT-DRGRP plasmid (Fig. 3g). These results suggested that KLC4 primarily affected all of the NHEJ and also the HR repair pathway after DNA damage.

### *KLC4* silencing induced CHK1/2 activation

**Fig. 4:**
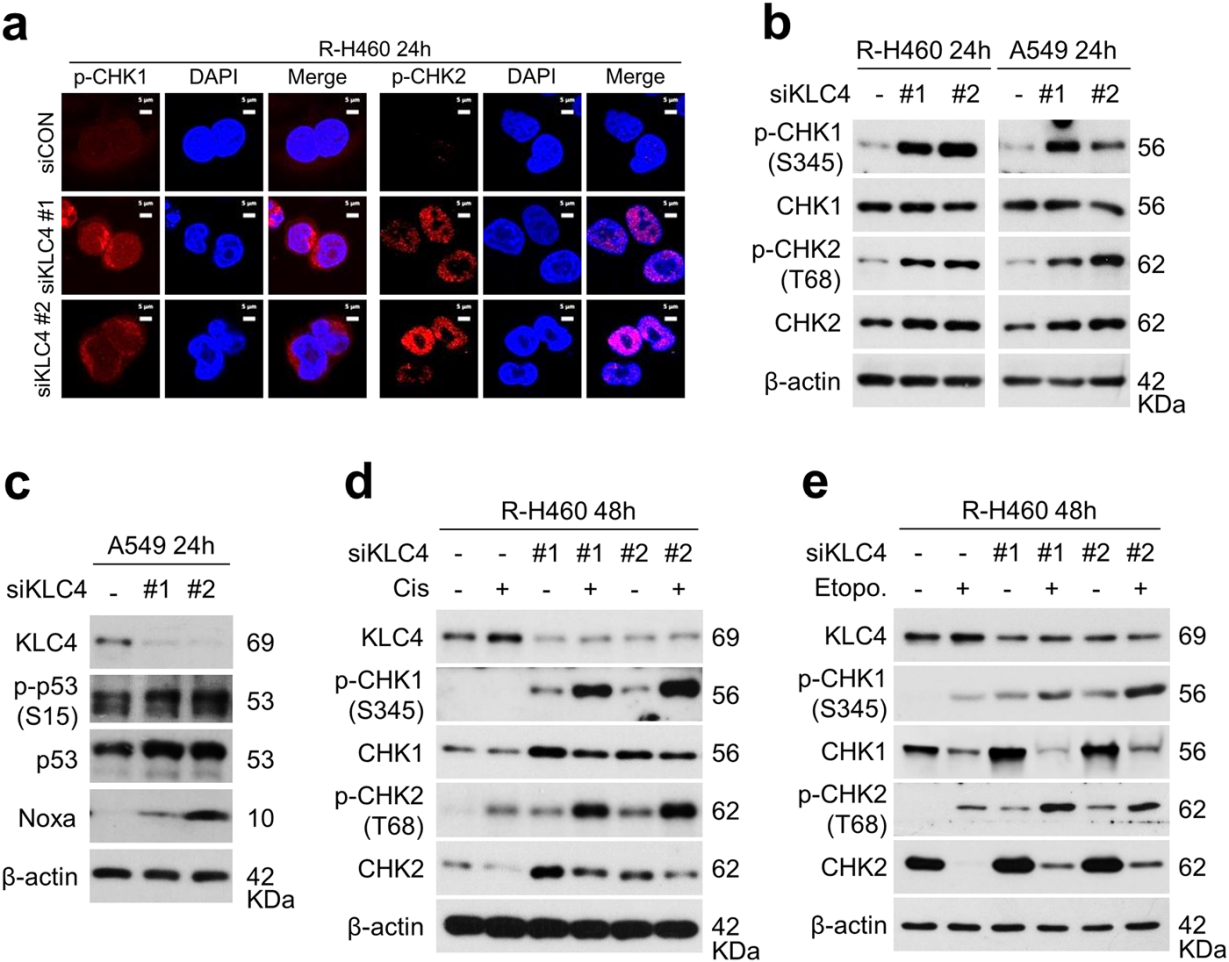
*KLC4* knockdown induced activation of CHK1/CHK2 in R-H460 cells. **a** Twenty-four hours after transfection with siCON (negative control) or siKLC4, the cells were fixed with paraformaldehyde and immunostained using antibodies targeting p-CHK1 and p-CHK2. **b** Cell lysates [from cells treated as in (a)] were prepared and used for immunoblotting with antibodies against p-CHK1 (S345), CHK1, p-CHK2 (T68), and CHK2. **c** Protein levels of KLC4, p-p53 (S15), p53, and Noxa, as determined using western blotting after transfection with *KLC4* siRNA. **d**, **e** Cells were treated with or without 10 μM cisplatin (**d**) or 10 μM etoposide (**e**) after transfection with *KLC4* siRNA. The cell lysates were used for immunoblotting with antibodies against KLC4, p-CHK1 (S345), CHK1, p-CHK2 (T68), and CHK2.

The above observations suggested that KLC4 may be related to the regulation of the DDR and/or repair of DNA damage. Therefore, we next investigated the effect of KLC4 on activation of CHK1 and CHK2 as the main effectors of DDR signaling^11^. Immunofluorescence observations showed no difference in the pattern of CHK2 and CHK1 phosphorylation after *KLC4* siRNA transfection, compared with that after transfection of negative control siRNA in R-H460 cells (Fig. 4a). This indicated that KLC4 may directly affect DDR signaling. Immunoblot analyses demonstrated that the levels of phospho-CHK1 and phospho-CHK2 were higher in *KLC4* siRNA-treated cells than in untreated cells (Fig. 4b). As these checkpoint kinases act upstream of p53^30^, we studied the phosphorylation status of p53 (Ser15) using western blot analysis; results showed that p53 phosphorylation increased after *KLC4* knockdown (Fig. 4c), whereas the total p53 level was not affected. Furthermore, compared with those induced by *KLC4* knockdown alone, the combination of *KLC4* knockdown and cisplatin/etoposide further increased the levels of phospho-CHK1 and phospho-CHK2 in R-H460 cells (Fig. 4d, e). Overall, these results indicated that KLC4 may prevent DNA damage repair and increase DNA damage by inhibiting CHK1/2 in lung cancer cells.

### Effect of KLC4 on CHK2-mediated apoptosis

**Fig. 5:**
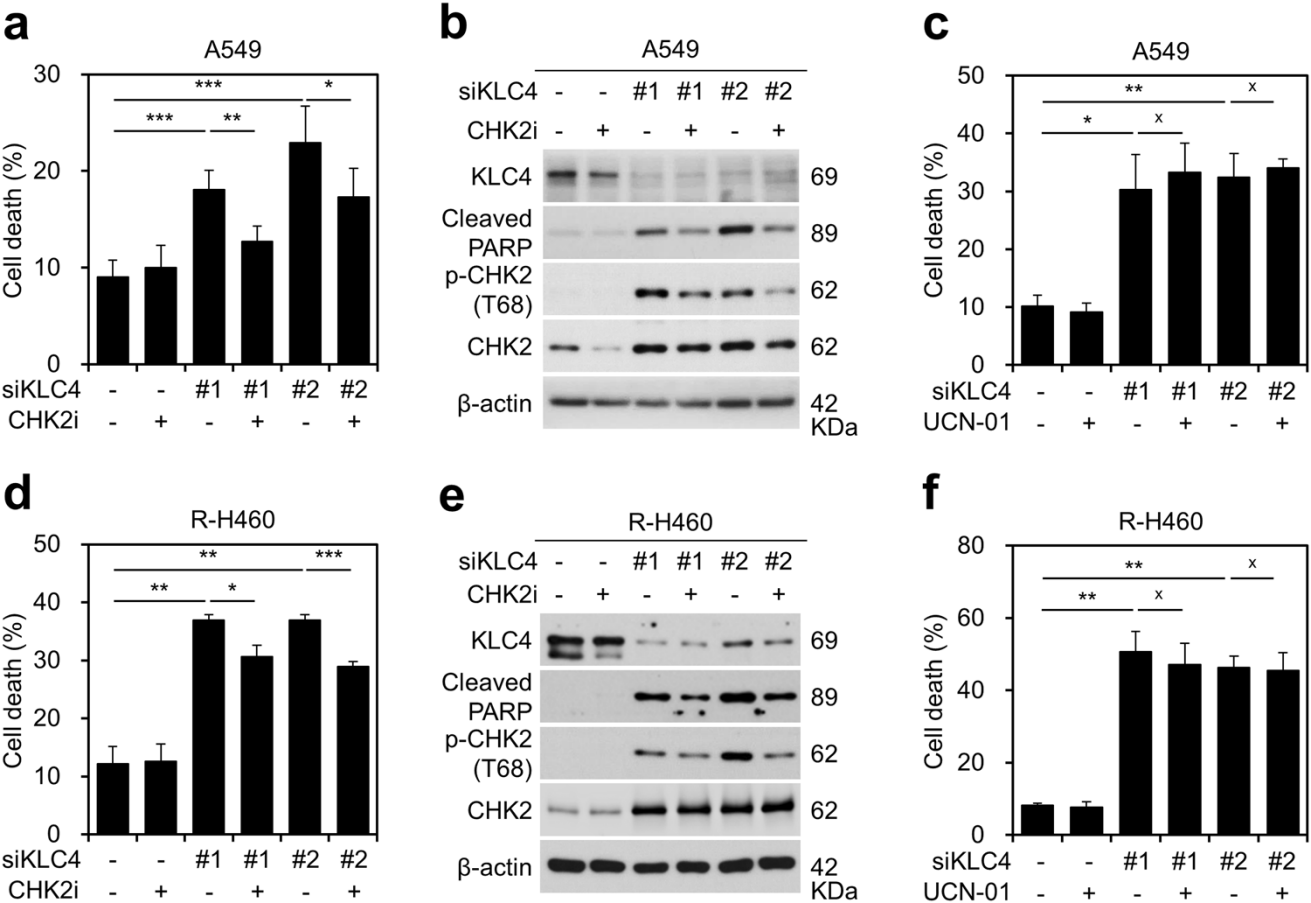
*KLC4* knockdown induced cell death via CHK2 activation. **a**, **b** A549 cells were treated with the CHK2 inhibitor (CHK2i), transfected with siCON or siKLC4, and incubated for 48 h. Cell death was determined using annexin V/propidium iodide (AV/PI) staining (**a**). Levels of the indicated proteins were determined using western blotting (**b**). **c** A549 cells were treated with the CHK1 inhibitor (UCN-01), transfected with siCON or siKLC4, and incubated for 48 h. Cell death was determined using AV/PI staining. **d** R-H460 cells were treated with the CHK2i after transfection with *KLC4* siRNA. Cell death in R-H460 cells was determined using AV/PI staining. **e** Protein levels of the indicated proteins were determined using western blotting after cells were treated as in (**d**). **f** R-H460 cells were treated with the CHK1 inhibitor UCN-01, transfected with *KLC4* siRNA, and incubated for 48 h; cell death was determined using AV/PI staining.

We next investigated whether KLC4 regulates these functions via CHK2 as a critical regulator of cell apoptosis and DNA repair^31^. Compared with that observed with siKLC4 treatment alone, the combination of *KLC4* knockdown and treatment with a CHK2 inhibitor resulted in a sharp reduction in apoptosis. The protein levels of cleaved PARP and phospho-CHK2 correlated in A549 and R-H460 cells (Fig. 5a, b, d, e). However, no further decrease in apoptosis was observed with depletion of *KLC4* and treatment with the CHK1 inhibitor (UCN-01) in A549 and R-H460 cells (Fig. 5c, f). These results showed that KLC4 controls DNA damage-mediated cell apoptosis via CHK2 and not CHK1.

### KLC4 correlated negatively with CHK2 and PRKDC in samples from patients with lung and colorectal cancer

**Fig. 6:**
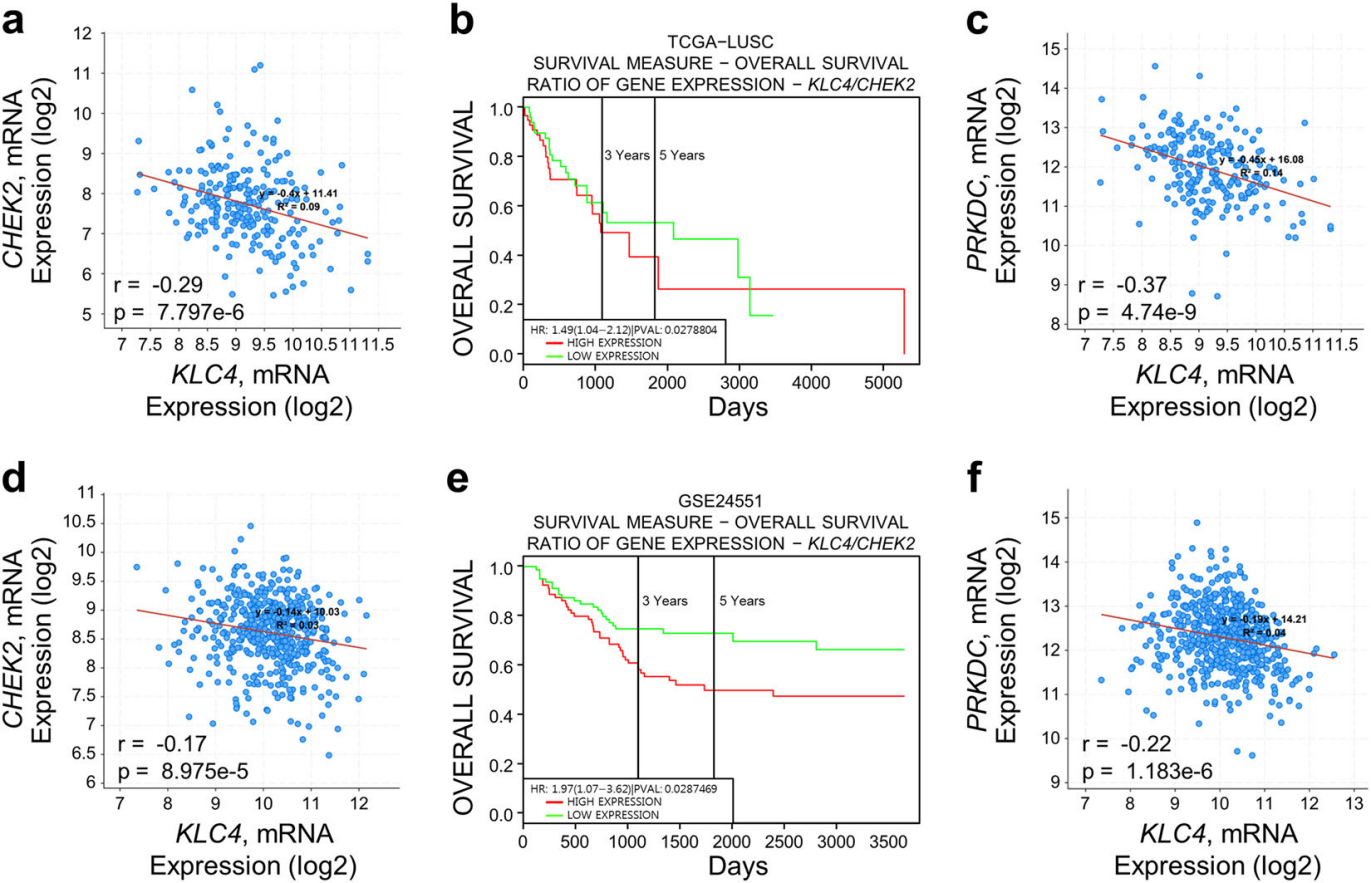
Correlation of *KLC4*-*CHEK2* signaling with the survival rate of patients with lung cancer. **a** Correlation of *KLC4* with *CHEK2* transcripts in the cBioportal datasets of patients with lung cancer (TCGA, Nature 2014). **b** Effects of *KLC4*/*CHEK2* expression on the overall survival of patients with lung cancer using Kaplan–Meier plotter analysis. **c** Correlation of *KLC4* with *PRKDC* transcript levels in the cBioportal datasets of patients with lung cancer. **d** Correlation of *KLC4* with *CHEK2* transcript levels in the cBioportal datasets of patients with colorectal cancer (TCGA, PanCancer Atlas). **e** Effects of *KLC4*/*CHEK2* expression on the overall survival of patients with colorectal cancer using Kaplan–Meier plotter analysis. **f** Correlation of *KLC4* with *PRKDC* transcript levels in the cBioportal datasets of patients with colorectal cancer.

It was confirmed that the expression of CHK1 and CHK2 was increased when *KLC4* siRNA was treated in R-H460 cells. Similarly, the mRNA levels of CHK1 and CHK2 were confirmed to have been affected from the transcription step. By confirming that KLC4 expression inhibition increases the mRNA levels of CHK1 and CHK2, it was found that KLC4 may be involved in the transcriptional step of CHKs (Supplementary Fig. 4a, b). In addition, the correlation between *KLC4* and *CHEK1*, *CHEK2* was investigated in both lung cancer and cervical cancer using RNA-sequencing data from publicly available microarray datasets. (Supplementary Fig. 4c and Fig. 6a, d).

Kaplan–Meier analysis showed that high *KLC4* and low *CHEK2* expression resulted in significantly lower survival rates for patients with lung and colorectal cancer (Fig. 6b, e). There was also a strong negative correlation between the *KLC4* mRNA and *CHEK2* or *PRKDC* (related to the NHEJ pathway; DNA-PKcs) mRNA levels in both patient groups (Fig. 6c, f). Together, these findings demonstrated the pivotal roles of KLC4, CHK2, and DNA-PKcs in regulating chemoresistance.

## Discussion

Previously, we had reported that *KLC4* depletion induces apoptosis of radioresistant cancer cells via mitochondrial dysfunction due to calcium ion influx. *KLC4* knockdown exhibited impaired mitochondrial respiration, increased mitochondrial reactive oxygen species production, and enhanced mitochondrial calcium uptake, which collectively led to mitochondrial dysfunction. For this reason, we proposed that KLC4, a kinesin superfamily member, may represent a novel and effective anticancer target, particularly for patients that exhibit radioresistance. Given that resistance to anticancer drugs, as well as radiation resistance, is a problem observed in many cancer patients, we aimed to determine whether *KLC4* affected anticancer resistance in this study. Our results showed that KLC4 regulated chemoresistance to cisplatin in NSCLC cells.

Adjuvant and neoadjuvant chemotherapy remain the main approaches of systemic treatment for many cancers at both early and advanced stages of the disease^32^. These treatments typically involve the use of DNA-damaging agents, as DNA integrity is important for suitable cell function and proliferation^33,34^. Cisplatin is a representative DNA-damaging agent; as an anthracycline antibiotic that intercalates into DNA, it hinders topoisomerase II progression, resulting in cytotoxicity mostly via inhibition of DNA replication and generation of DSBs^33^. However, resistance (innate and/or acquired) to cisplatin is common in the treatment of many types of cancer^35^. Therefore, it is important to understand the cellular mechanisms related to chemoresistance for identifying novel therapeutic targets that may prevent acquisition of drug resistance.

Based on the standardized nomenclature by the society of kinesin researchers, the human kinesin superfamily has 14 members^36^. However, the nomenclature of kinesin is ambiguous, resulting in several designations and redundant names for a single kinesin. Individual kinesins play an essential role in the intracellular transport of membranous organelles for regulating morphogenesis and ensuring proper function of the cell and protein complexes in a microtubule- and ATP-dependent manner. Furthermore, kinesins have been shown to play vital roles in many cellular functions regarding cell division and membrane trafficking events, including endocytosis and transcytosis^37–39^. Although members of the kinesin family vary in shape, a typical kinesin occurs as a protein dimer (molecular pair) consisting of two heavy chains and two light chains^40^. KLC4 belongs to the kinesin light chain family and is a microtubule-associated force-producing protein that has been suggested to play a critical role in organelle transport^40,41^. The light chain may function in coupling of cargo to the heavy chain or in the modulation of its ATPase activity^36^. However, to the best of our knowledge, this is the first study to focus on the biological chemoresistance function of KLC4 in cancer. Functions of KLC4 have not been extensively reported; however, Li et al. recently reported that SETD3 reduces KLC4 expression to improve the sensitization of cervical cancer cells to radiotherapy, and that SETD3 down-regulates *KLC4*, thus contributing to the radiosensitivity of cervical cancer cells. Hence, it can be inferred that KLC4 overexpression can abolish the regulatory role of SETD3^42^. This result, in turn, is consistent with the finding that *KLC4* is involved in radioresistance.

Our results showed that up- or downregulation of *KLC4* can affect cancer cell proliferation. Expression of KLC4 was also related to the sensitivity of lung cancer cells to chemotherapy. Thus, inhibiting KLC4 may be a new strategy for overcoming chemoresistance in lung cancer cells and simultaneously enhancing the effects of chemotherapy in patients with lung cancer. Furthermore, we demonstrated that these effects of KLC4 are mediated via regulation of CHK2, which affects the DNA-damaging action of chemotherapeutic drugs. Several studies have shown that CHK2 plays an important role in the maintenance of G2/M arrest and induction of apoptosis in response to radiation therapy or chemotherapy, as lack of CHK2 compromised the G2/M checkpoint and apoptosis, subsequently resulting in chemoresistance^11,31,43–45^. In agreement with the results of these previous reports, we observed that depletion of *KLC4* resulted in CHK1/2 activation. However, knockdown of *KLC4* in cells treated with the CHK1 inhibitor had no further effects on chemoresistance. Thus, we suggested that *KLC4* siRNA treatment induced cell death via CHK2, and not CHK1, activation.

Overall, our observations show that the KLC4-CHK2 axis might act as a potential target for developing novel anticancer therapeutics and as a candidate biomarker of tumor chemoresistance. These independent observations showed that a comprehensive analysis of the expression levels of chemoresistance-related genes in lung cancer cells may be advantageous in predicting the cellular chemoresistance and cross-resistance to chemotherapeutic agents, which may improve selection of effective drugs for therapy of patients with lung tumors. In this regard, the present study provides new insights into the crosstalk between the CHK2 pathway and the KLC4-regulated apoptotic pathway, which contributes to our understanding of the mechanism underlying the development of drug resistance in cancer cells. The mechanism by which KLC4 can induce the expression of CHK1 and CHK2 is planned to be studied in the future, to determine whether or not mitochondrial dysfunction or other functions affect KLC4. KLC4 is a protein that belongs to the light chain of the motor protein kinesine, which, in turn, is involved in the binding of cargo (protein) carried by the motor protein. This suggests that CHK1/2 may be involved in the activation of the proteins involved in the activation of CHK1/2. We are conducting further studies to identify the binding protein of KLC4. Furthermore, this study indicates that treatment regimens should be planned according to the specific characteristics and genetic background of each patient, highlighting the need for identifying effective biomarkers for cisplatin resistance.

In conclusion, we propose that chemotherapy might be more effective when combined with RNAi-mediated knockdown of *KLC4*. For practical applications of this therapeutic strategy, a good vector system has to be developed and tested. In addition, investigations on the effects of KLC4 inhibition on normal cells are required to minimize any potential complications of its inhibition in clinical application for lung cancer treatment.

## Materials and methods

### Cell culture and treatment

Human lung cancer (A549, H460) cell lines were purchased from the American Type Culture Collection (Manassas, VA, USA). The lung cancer cells were cultured in Roswell Park Memorial Institute (RPMI)-1640 medium supplemented with 10% fetal bovine serum and 1% antibiotic-antimycotic. The radioresistant cell line R-H460, derived from parental radiosensitive H460 lung cancer cells cumulatively administered 2-Gy radiation twice a week for 20 weeks, were also used^20^. The direct repeat-green fluorescent protein (DR-GFP) and end-joining (EJ)-GFP-expressing reporter cell lines derived from the U2OS cells transfected with pHRPT-DRGRP and pimEJ5GFP plasmids, respectively, were provided by Dr. Jae-Hoon Ji (Ajou University School of Medicine, Suwon, South Korea). The cells were irradiated using a 137Cs source (Atomic Energy of Canada, Ltd., Canada) at a dose rate of 3.81 Gy/min and treated with cisplatin, etoposide, gefitinib, and Taxol (Sigma, St. Louis, MO, USA).

### Evaluation of apoptosis using annexin V/propidium iodide staining

In order to quantify cell death caused by experimental treatments as mentioned above, the cells were seeded at a density of 3 × 10^5^ cells per 60-mm dish and subjected to treatments, as described in experimental conditions. After being incubated in accordance with experimental conditions, trypsin-EDTA was treated with the cells and then washed with phosphate buffered saline. Cells were transferred to lx binding buffer before being incubated with Annexin V-FITC and PI-PE and analyzed by FACScan flow cytometer (BD Biosciences, San Jose, CA, USA).

### Cell viability assay

The cells were seeded at 5000 cells/well in a 96-well plate, subjected to various treatments as mentioned above, and incubated for 24 h. In order to quantify cell viability, Cyto XTM reagent (LPS solution) was treated for 2 h and then quantified using Multiscan EX (Thermo) at 450 nm.

### Immunofluorescence confocal microscopy

Immunofluorescence staining for KLC4 (Sigma, St. Louis, MO, USA), γH2AX3 [a marker of DNA double-stranded breaks (DSBs); Merck KGaA, Darmstadt, Germany], p-CHK1(S345), and p-CHK2(T68) (Cell Signaling Technology Inc., Beverly, MA, USA) was performed as described previously^46^. Cell nuclei were identified by staining with 4,6-diamidino-2-phenylindole.

### Western blot analysis

Western blot analyses were performed as described previously^47^ using primary antibodies against KLC4, β-actin (Sigma, St. Louis, MO, USA), γH2AX (Merck KGaA, Darmstadt, Germany), p-p53 (S15), p53, Noxa (Santa Cruz Biotechnology Inc., USA), p-CHK1(S345), CHK1, p-CHK2(T68), CHK2, cleaved poly(ADP-ribose) polymerase (Asp214), and active caspase-3 (Cell Signaling Technology Inc., Beverly, MA, USA).

### Knockdown of proteins by siRNA

As described by the manufacturer (Genolution, Seoul, Korea), the following human-specific siRNAs synthesized were used: siKLC4 #1, 5′-CCG UUC UAU GGA AAA CAU UUU-3′ (sense) and 5′-AAU GUU UUC CAU AGA ACG GUU-3′ (antisense); siKLC4 #2, 5′-CCA GAA UAA GUA UAA GGA AUU-3′ (sense) and 5′-UUC CUU AUA CUU AUU CUG GUU-3′ (antisense). The cells were transfected with 40 nM siRNA using lipofectamine RNAimax based on each experimental condition.

### Colony forming assay

Cell survival was determined by Colony forming assays. Put succinctly, cells were seeded into triplicate 60-mm tissue culture dishes at densities of 4 × 10^2^ cells / dish. Meanwhile, *KLC4* siRNA and cisplatin were treated on the cells. After a period of 14 days, colonies generated from viable cells were stained with trypan blue solution, counting of which was done using a colony counter (Imaging Products, Chantilly, VA).

### HR and NHEJ repair assays

The U2OS DR-GFP and EJ-GFP stable reporter cells were transfected with small interfering RNA (siRNA) targeting *KLC4* to knockdown its expression. Human-specific siRNAs were synthesized as per the manufacturer’s instructions (Genolution, Seoul, Korea) and used as described previously^21,47^. At the same time, U2OS (DR-GFP, EJ-GFP) reporter cells were transfected with the pCB-ASce plasmid by Prf. Yoon Sil Lee (Ewha Womens University School of Pharmacy, Seoul, South Korea) using Mirus2020 reagent (Mirus Bio LLC, USA) according to the manufacturer’s instructions. The GFP-positive cells were counted using flow cytometry analysis 48 h post-transfection.

### Quantitative reverse transcription-PCR (RT-qPCR) analysis

As described previously^20^, the Quantitative reverse transcription-PCR (RT-qPCR) was performed using the following primer pairs: *CHEK1*, 5′-CGGTATAATAATCGTGAGCG-3′ (sense) and 5′-TTCCAAGGGTTGAGGTATGT-3′ (antisense); *CHEK1*, 5′-GCGCCTGAAGTTCTTGTTTC-3′ (sense) and 5′-GCCTTTGGATCCACTACCAA-3′ (antisense); as well as *GAPDH*, 5′-CATCTCTGCCCCCTCTGCTGA-3′ (sense), and 5′-GGATGACCTTGCCCACAGCCT-3′ (antisense).

### Data mining using the Kaplan–Meier plotter database and cBioportal database

Kaplan–Meier survival curves related to *KLC4*/*CHEK2* expression were generated for patients with lung cancer and colorectal cancer. Data were analyzed using the KM-plotter (http://genomics.jefferson.edu/proggene/). Coexpression data for *KLC4, CHEK1, CHEK2*, and *PRKDC* were extracted from The Cancer Genome Atlas RNA-sequencing data in the cBioportal database (https://www.cbioportal.org).

### Statistical analyses

Cell culture experiments were repeated at least thrice. All data are shown as the mean ± standard deviation. Statistical differences between groups were evaluated using Student’s *t* test and *P* value < 0.05 was considered significant.

### Grants

This study was supported by a grant from the Korea Institute of Radiological and Medical Sciences, which was funded by the Ministry of Science, ICT, and Future Planning, Republic of Korea [grant number: 50531-2020, 50538-2020].

## Supplementary information

supple fig1

supple fig2

supple fig3

supple fig4

supple fig legend
