# Supplementary figures and images for: Kinesin light chain 4 as a new target for lung cancer chemoresistance via targeted inhibition of checkpoint kinases in the DNA repair network
Source: Cell Death Dis. 2020 May 26;11(5):398. doi: 10.1038/s41419-020-2592-z (PMC7250887; doi:10.1038/s41419-020-2592-z)

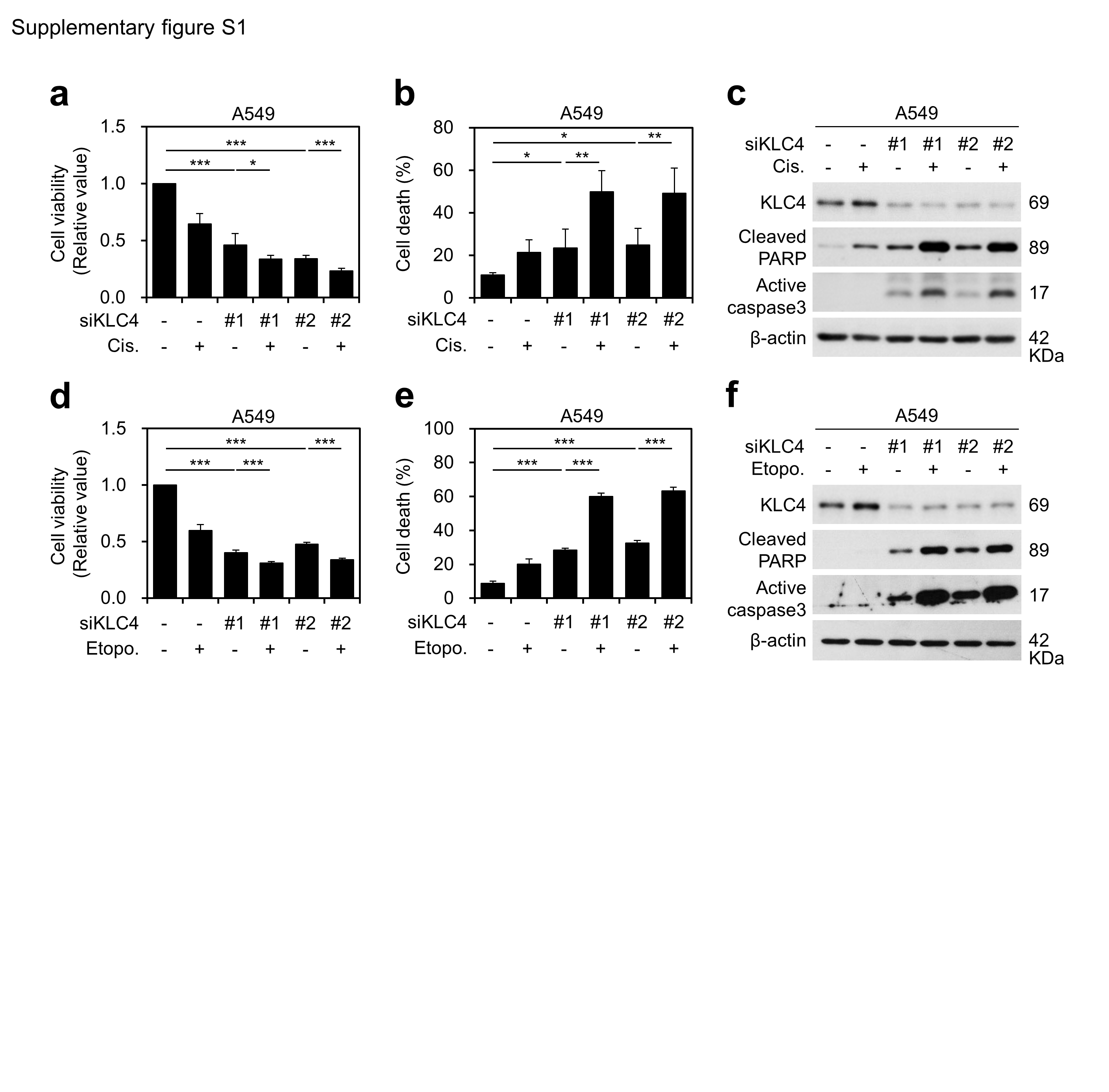

Supplement: Supplementary file 1 — supple fig1 [file 41419_2020_2592_MOESM1_ESM.tif]

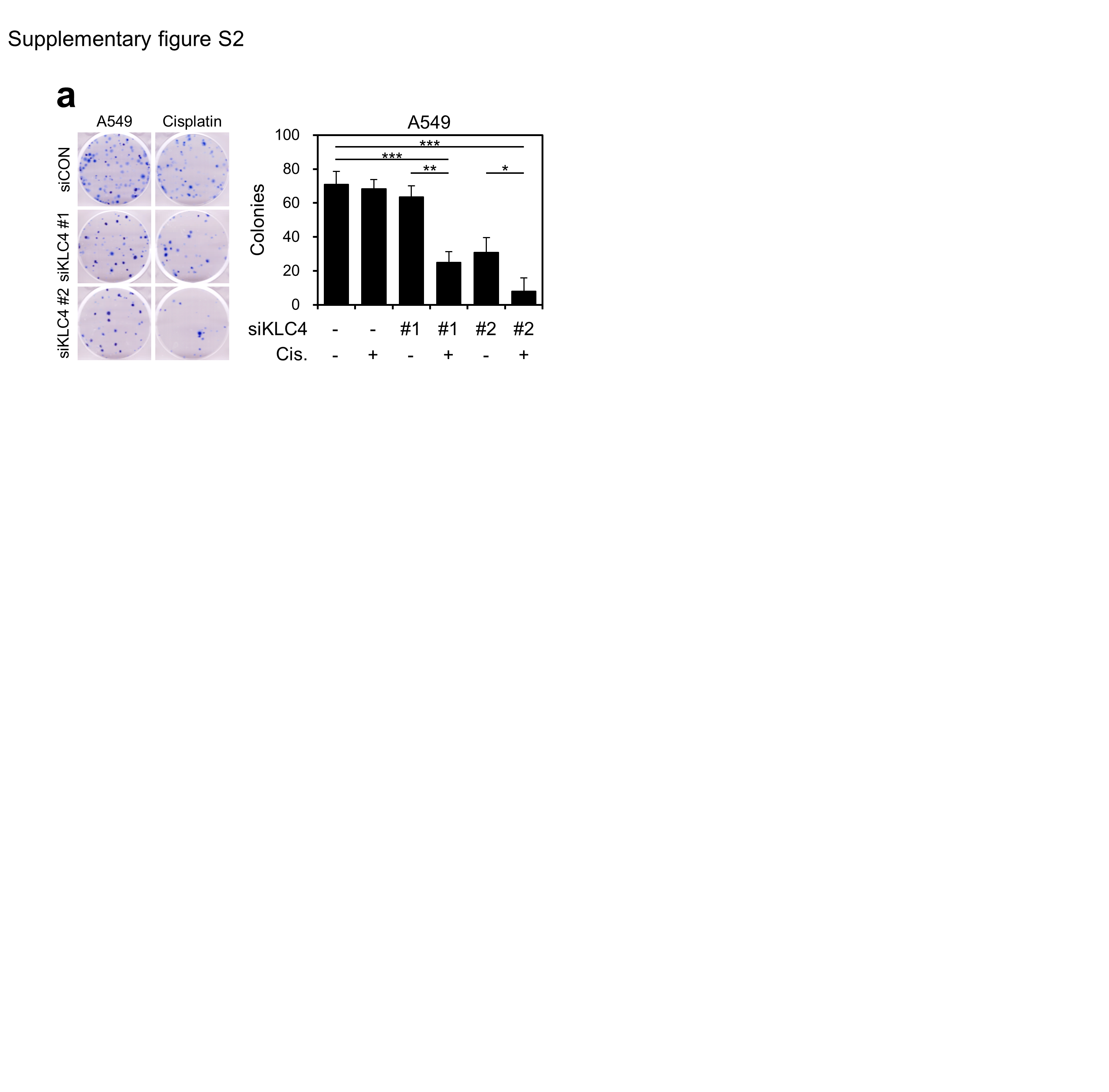

Supplement: Supplementary file 2 — supple fig2 [file 41419_2020_2592_MOESM2_ESM.tif]

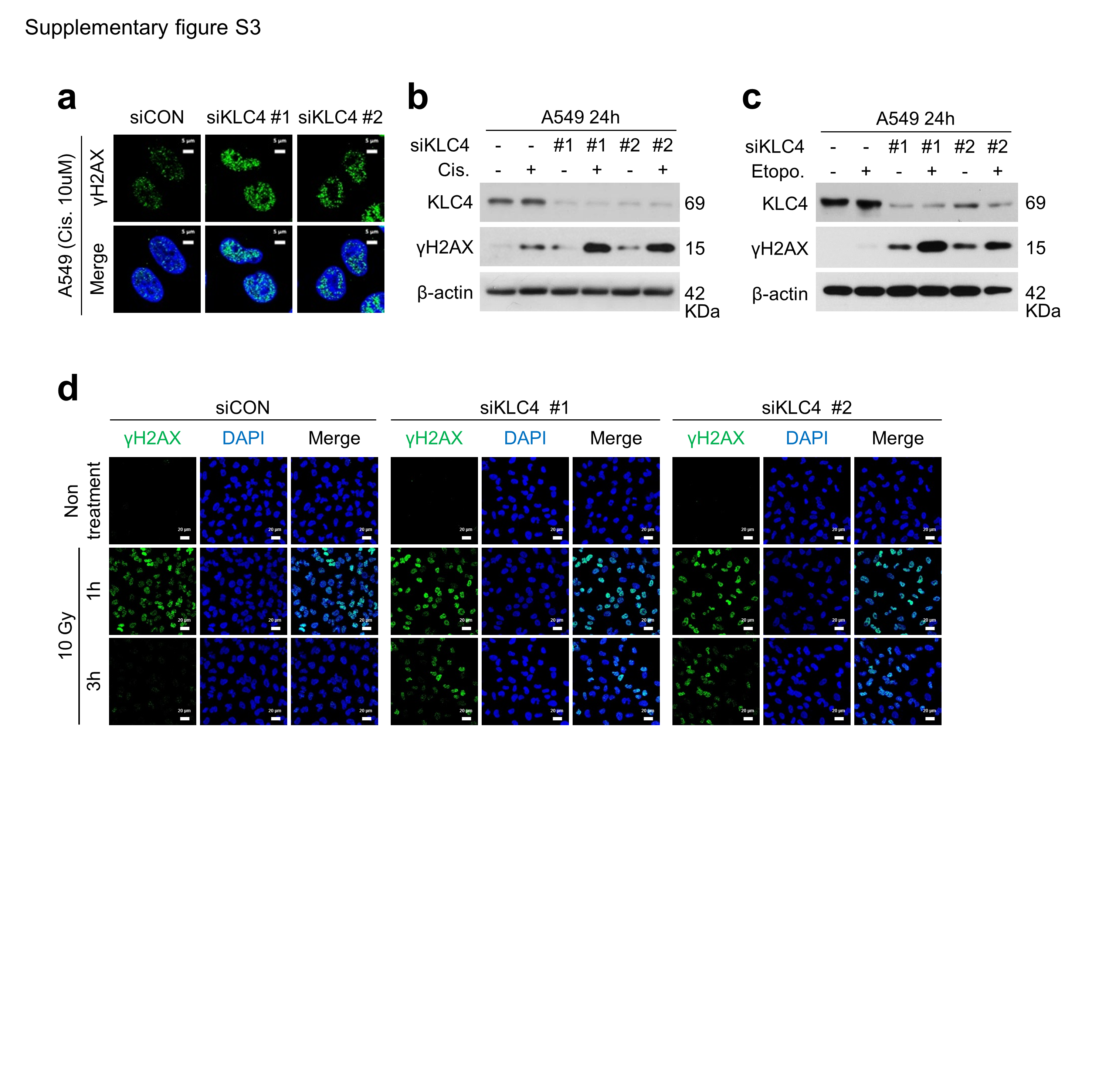

Supplement: Supplementary file 3 — supple fig3 [file 41419_2020_2592_MOESM3_ESM.tif]

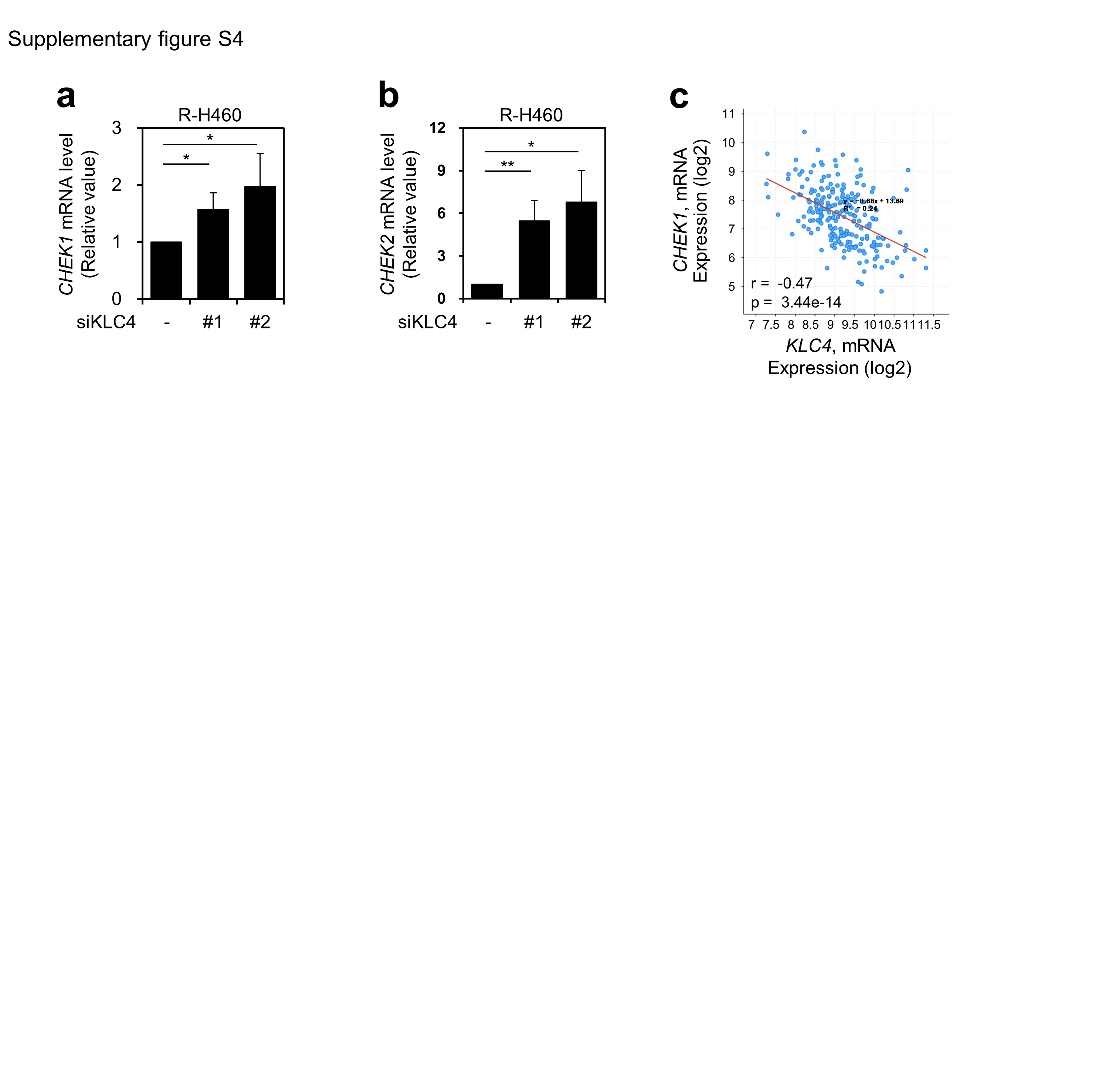

Supplement: Supplementary file 4 — supple fig4 [file 41419_2020_2592_MOESM4_ESM.tif]
