# Supplementary material for: Kinesin light chain 4 as a new target for lung cancer chemoresistance via targeted inhibition of checkpoint kinases in the DNA repair network
Source: Cell Death Dis. 2020 May 26;11(5):398. doi: 10.1038/s41419-020-2592-z (PMC7250887; doi:10.1038/s41419-020-2592-z)
Supplement: Supplementary file 5 — supple fig legend [file 41419_2020_2592_MOESM5_ESM.docx]

**Supplementary figure S1** (a) Analysis of the viability of A549 cells treated with or without 10 μM cisplatin after transfection with siCON or siKLC4. (b) Determination of cell death in A549 cells (treated as in a) by AV/PI staining. (c) Protein levels of KLC4, cleaved PARP, and active caspase-3 (cell death marker) as determined by western blotting. (d) A549 cells were treated with or without 10 µM Etoposide after transfection with *KLC4* siRNA. (e-f) Cell death was measured 48 h after treatment by cell viability assay, AV/PI staining (e) and western blotting (f).

**Supplementary figure S2** (a) KLC4 knock-down cells were untreated or were treated with 10 μM cisplatin for 14 d. Colony formation was visualized by trypan blue staining.

**Supplementary figure S3** (a) A549 cells were treated with 10 µM cisplatin after transfection with *KLC4* siRNA for 24h. Cells were fixed and immunostained using antibody targeting γH2AX. (b-c) A549 cells were pre-treated with 10 µM cisplatin (b) and 10 µM etoposide (c), and after transfection with siKLC4, cell lysates were prepared and used for immunoblotting with antibodies against KLC4 and γH2AX. (d) R-H460 cells were transfected with 10Gy irradiation after transfection with *KLC4* siRNA for 24h. Cells were fixed and immunostained using antibody targeting γH2AX.

**Supplementary figure S4** (a-b) R-H460 cells were transfected with siCON or the indicated concentration of siKLC4 for 24 h. *CHEK1*(a) and *CHEK2*(b) mRNA levels were determined via RT-qPCR. (c) Correlation of *KLC4* with *CHEK2* transcripts in the cBioportal datasets of patients with lung cancer [(TCGA, Nature 2014)](https://www.cbioportal.org/study?id=luad_tcga_pub).
